# Une hydrocèle du canal de Nuck découverte au cours d´un bilan d´infertilité: à propos d’un cas atypique

**DOI:** 10.11604/pamj.2021.38.321.27252

**Published:** 2021-04-01

**Authors:** Jihane Habi, Amal Rami, Hind Guerroum, Mariam Kassimi, Nabil Chikhaoui, Mohamed Mahi

**Affiliations:** 1 Department of Radiology, Faculty of Medicine, Mohammed VI University of Health Sciences/Cheikh Khalifa International University Hospital, Casablanca, Morocco

**Keywords:** Imagerie, hydrocèle, canal de Nuck, à propos d’un cas, Imaging, hydrocele, canal of Nuck, case report

## Abstract

L´hydrocèle du canal de Nuck est une pathologie rare de la femme, souvent découverte durant l´enfance. Cette présentation rapporte un cas rare d´hydrocèle découverte à l´âge adulte au cours d´un bilan d´infertilité. A l´admission au service, la patiente a rapporté une tuméfaction inguinale droite chronique non douloureuse. L´imagerie a été typique d´une hydrocèle du canal de Nuck. L´échographie a retrouvé une formation kystique avec quelques fin septas et l´'imagerie par résonance magnétique (IRM) n´a pas retrouvé de communication avec le péritoine. Avec les mêmes moyens d´imagerie, un utérus bicorne non cloisonné a été découvert pouvant rentrer dans le cadre de son bilan d´infertilité. Le but principal de ce travail est de faire connaitre l´imagerie typique de l´hydrocèle du canal de Nuck un diagnostic peu connu et de le faire intégrer parmi les diagnostics différentiels des tuméfactions inguinales chez la femme.

## Abstract (en)

Hydrocele of the canal of Nuck is a rare woman condition often detected during childhood. We here report a rare case of hydrocele detected in adulthood during infertility evaluation. The patient presented with chronic non painful right inguinal swelling. Imaging test demonstrated typical image of hydrocele of the canal of Nuck. The ultrasound found cyst formation with few thin septa and MRI did not find any communication with the peritoneum. The same imaging tests were performed which showed non-partitioned bicornuate uterus that could fall under the framework of infertility evaluation. The main purpose of this study was to report the typical imaging features of hydrocele of the canal of Nuck, which is a little known diagnosis that should be integrated into differential diagnoses of inguinal swellings in women.

## Introduction

L´hydrocèle se constitue par la persistance de l´ouverture du canal de Nuck au cours de la première année de vie. C´est une pathologie congénitale féminine rare. Elle est souvent découverte au bas âge par une tuméfaction inguinale. Sa prévalence ne peut être précisément estimée à cause du nombre limite des cas et des séries rapportées [1]. La découverte de cette pathologie est rare à l'âge adulte et souvent de façon fortuite. D'où la présentation de notre cas de l'hydrocèle du canal de Nuck découverte accidentellement lors d'un bilan d'infertilité chez une femelle adulte. A travers ce travail, nous nous concentrons sur l'hydrocèle du canal de Nuck. Nous présentons les résultats de l'imagerie conduisant à son diagnostic positif et à sa caractérisation.

## Patient et observation

Le cas concerne une femme nullipare âgée de 28 ans, qui s´était présentée à notre service pour un bilan d'infertilité. Elle avait comme antécédent un angiome veineux de l´avant-bras opéré. À l'examen clinique, la patiente a rapporté une tuméfaction inguinale droite indolore progressant depuis quatre ans sans notion de traumatisme inguinal ou trouble digestif associé. A la palpation, la tuméfaction était mobile et irréductible. En se basant sur ces données cliniques, les diagnostics suivants ont été évoqués; hernie inguinale, hydrocèle du canal de Nuck, tumeurs des tissus mous, ganglions lymphatiques et anévrisme.

**Figure 1:**
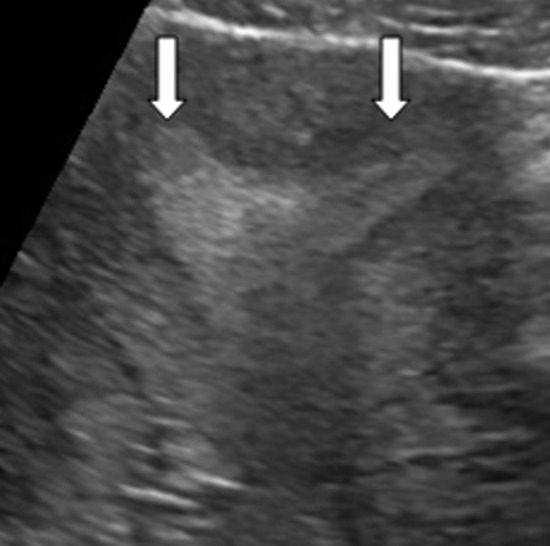
échographie; utérus bicorne malformative (flèche)

**Figure 2:**
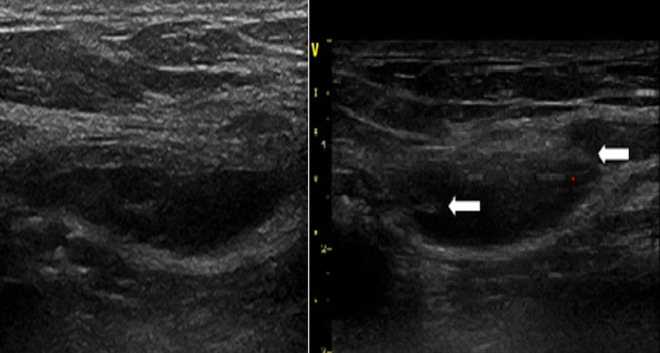
échographie; hydrocèle anéchogène avec de fines cloisons (flèche)

**Figure 3:**
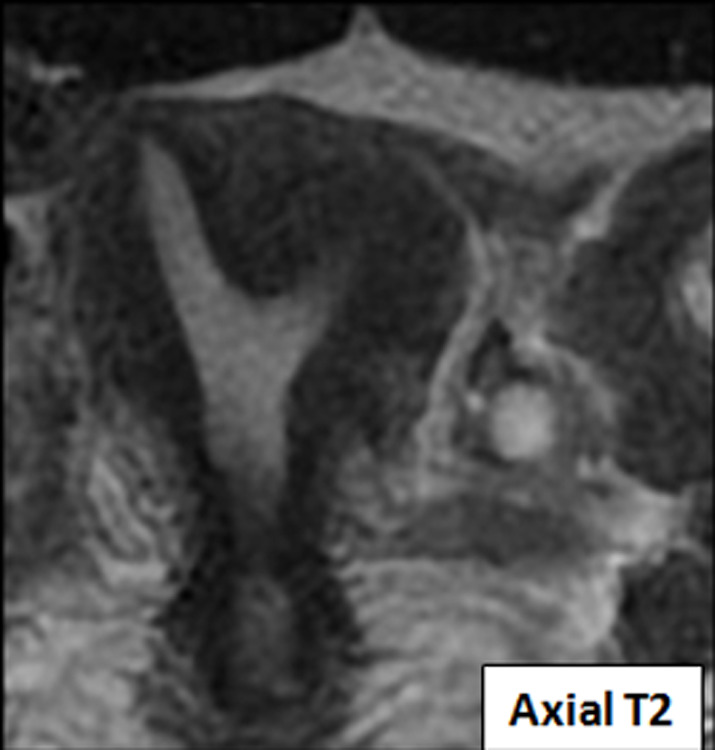
IRM; coupe en axial T2 montrant un utérus bicorne uni cervical non cloisonné

Dans le cadre du bilan d'infertilité, une échographie a été indiquée et réalisée en premier lieu. Elle a d'abord révélé une malformation utérine bicorne (Figure 1). Puis, une formation kystique inguinale droite, anéchogène, en forme de saucisse, bien limitée, contenant quelques fines cloisons, sans augmentation de volume après la manœuvre de Valsalva (Figure 2). L'IRM pelvienne a été indiquée pour compléter la caractérisation. Elle a mis en évidence un utérus uni cervicale bicorne partiel (Figure 3).

**Figure 4:**
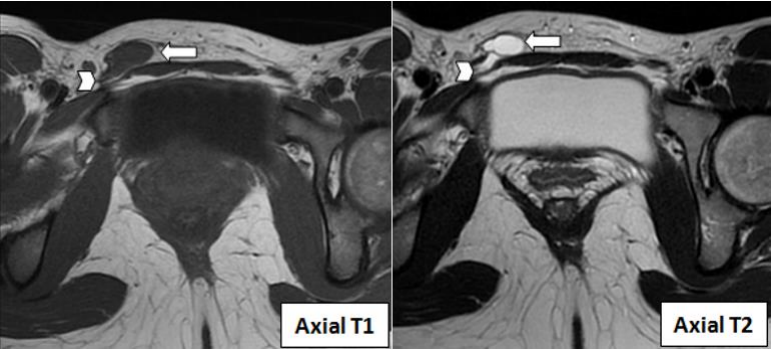
IRM en coupe axiale montrant l´hydrocèle du canal de Nuck en hyposignal T1, hypersignal T2 avec quelques cloisons (flèche)

**Figure 5:**
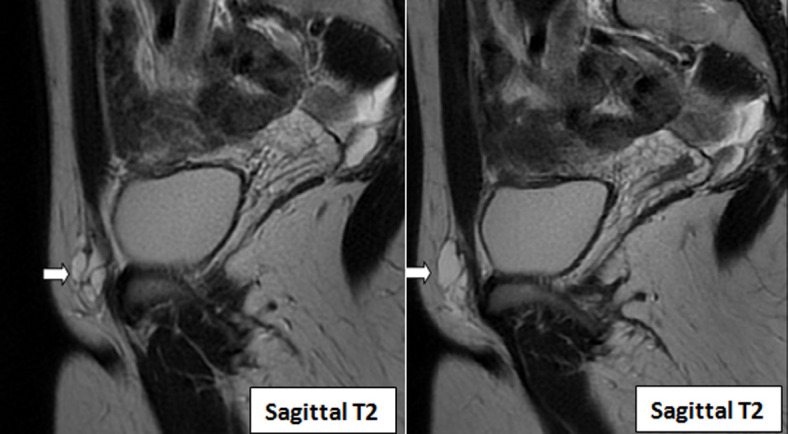
IRM en différentes coupes sagittales T2 révélant un kyste du canal de Nuck multi lobulé cloisonné (flèche)

Quant à la formation inguinale, elle était en hypo signal T1, hypersignal T2, mesurée à 2cmx2cm, avec quelques cloisons, et sans communication avec le péritoine (Figure 4 et Figure 5). Devant ces résultats d'imagerie, nous avons retenu le diagnostic de l'hydrocèle du canal de Nuck associée à une malformation utérine. L'exérèse chirurgicale est le traitement de référence pour tout type d'hydrocèle du canal Nuck. Cependant, notre patiente a préféré ne pas être opérée car le kyste était limite asymptomatique et sans vrai rapport avec son infertilité. À notre connaissance, la patiente se porte bien, sans saignement ni complications infectieuses et elle continue ses investigations dans le bilan d´infertilité.

## Discussion

**Figure 6:**
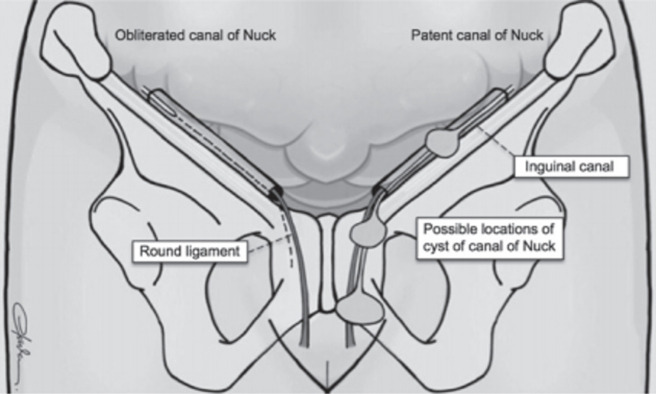
anatomie du canal inguinal avec un canal de Nuck physiologiquement fermé à gauche; à droite: présentations des différents potentiels sites du kyste à travers le canal lorsqu'il reste perméable

Le canal de Nuck, nommé d'après l'anatomiste néerlandais Anton Nuck en 1691 [2] est une petite partie du péritoine pariétal qui passe avec le gubernaculum à travers le canal inguinal (Figure 6). Le canal de Nuck disparait normalement au cours de la première année de vie. Son obturation se déroule progressivement du haut vers le bas [3, 4]. Lorsque le canal de Nuck reste perméable, cela conduit à la formation de l'hydrocèle. Le diagnostic de cette pathologie est rarement découvert fortuitement chez la femme adulte, comme il est le cas de notre patiente lors de son bilan d'infertilité.

L'hydrocèle du canal de Nuck est classée en 3 types: le type le plus fréquent est l'hydrocèle enkystée, suivie de l'hydrocèle communicante, et enfin les hydrocèles biloculaires [5, 6]. Cette pathologie peut être retrouvée dans différents sites à travers le canal inguinal (Figure 6).

Le kyste du canal de Nuck présente une symptomatologie locale, décrite comme une masse légèrement douloureuse irréductible ou réductible dans la région inguinale qui s'étend généralement aux grandes lèvres et n´augmente pas de volume à la manœuvre de Valsalva. Il existe plusieurs diagnostics différentiels de l´hydrocèle: hernie inguinale, anévrisme, ganglions lymphatiques et tumeurs des tissus mous. La principale est une hernie inguinale car elle est associée à un tiers des cas.

L´imagerie joue un rôle diagnostic principal. L´échographie est le premier examen à réaliser. Il est anodin, abordable et fournit des détails sur la localisation, le contenu et la taille du kyste. Dans la littérature, l'aspect échographique de l'hydrocèle du canal de Nuck est celui d´une structure kystique anéchogène, à paroi mince, bien définie, de forme tubulaire ou en « saucisse », ou même d´apparence multi kystique [7]. La manœuvre de Valsalva en échographie peut faire la différence entre l'hydrocèle et la hernie inguinale, à travers le contenu échogène (intestin, ovaire ou mésentérique) et l´augmentation du volume de la hernie au cours de cette manœuvre. Au Doppler couleur, le kyste du canal de Nuck ne montre pas de vascularisation interne [8].

En ce qui concerne l´IRM, elle se réalise dans le but diagnostic lorsque les résultats échographiques sont non concluants. L´hydrocèle apparaît généralement comme une lésion kystique à parois fines, en hypo intense en T1 et en hyperintense en T2, avec quelques fines cloisons comme l´imagerie de notre patiente. L´IRM permet également de réaliser le bilan pré chirurgical [9], sa haute résolution spatiale offre une meilleure analyse et visibilité des rapports du kyste du canal de Nuck avec les structures intra péritonéales.

Le traitement de choix de toutes les hydrocèles du canal de Nuck est une exérèse chirurgicale pour éviter toute complication infectieuse ou hémorragique [6-10]. Cependant, notre patiente n'a pas voulu être opérée, étant donné que l'hydrocèle du canal de Nuck était asymptomatique et non causale de son infertilité.

## Conclusion

L'hydrocèle du canal de Nuck est une pathologie féminine rare. Toute tuméfaction inguinale doit entraîner la recherche d'un kyste du canal de Nuck quel que soit l'âge de la patiente. L´imagerie joue un rôle principal dans la prise en charge diagnostique et pré thérapeutique. L'échographie est l'examen de choix pour poser le diagnostic positif. L´IRM reste l'examen complémentaire en cas de difficulté diagnostique et pour le bilan anatomique pré-chirurgical détaillé.

## Remerciements

On aimerait remercier toute l´équipe de Anastasia Prodromidou, Anna Paspala, Dimitrios Schizas, Eleftherios Spartalis, Constantinos Nastos, Nikolaos Machairas pour nous avoir donné leur permission d´illustrer par une figure de leur article “Cyst of the Canal of Nuck in adult females: a case report and systematic review”.
